# Self-Assembly of Rhein and Matrine Nanoparticles for Enhanced Wound Healing

**DOI:** 10.3390/molecules29143326

**Published:** 2024-07-15

**Authors:** Xunxun Wu, Ranqing Zang, Yiting Qiu, Ni Yang, Meiyan Liu, Site Wei, Xianxiang Xu, Yong Diao

**Affiliations:** School of Medicine, Huaqiao University, Quanzhou 362021, China; wuxunxun2015@163.com (X.W.); 13612820860@163.com (R.Z.); qytdezh@163.com (Y.Q.); 2134111024@stu.hqu.edu.cn (N.Y.); 15863607363@163.com (M.L.); wei5826013@163.com (S.W.); xuxianxiang@hqu.edu.cn (X.X.)

**Keywords:** self-assembly, nanoparticle, Rhein, Matrine, hydrogel, wound healing

## Abstract

Carrier-free self-assembly has gradually shifted the focus of research on natural products, which effectively improve the bioavailability and the drug-loading rate. However, in spite of the existing studies, the development of self-assembled natural phytochemicals that possess pharmacological effects still has scope for further exploration and enhancement. Herein, a nano-delivery system was fabricated through the direct self-assembly of Rhein and Matrine and was identified as a self-assembled Rhein-Matrine nanoparticles (RM NPs). The morphology of RM NPs was characterized by TEM. The molecular mechanisms of self-assembly were explored using FT-IR, ^1^H NMR, and molecular dynamics simulation analysis. Gelatin methacryloyl (GelMA) hydrogel was used as a drug carrier for controlled release and targeted delivery of RM NPs. The potential wound repair properties of RM NPs were evaluated on a skin wound-healing model. TEM and dynamic light scattering study demonstrated that the RM NPs were close to spherical, and the average size was approximately 75 nm. ^1^H NMR of RM NPs demonstrated strong and weak changes in the interaction energies during self-assembly. Further molecular dynamics simulation analysis predicted the self-assembly behavior. An in vivo skin wound-healing model demonstrated that RM NPs present better protection effect against skin damages. Taken together, RM NPs are a new self-assembly system; this may provide new directions for natural product applications.

## 1. Introduction

The emergence of a carrier-free self-assembly delivery system can effectively improve the bioavailability of drugs and increase the drug loading rate [1]. Carrier-free self-assembled nano-drugs are a kind of nano-drug delivery system, which does not depend on traditional drug carriers, such as macromolecules [2,3]. These drug molecules usually have special hydrophilic and hydrophobic regions, which enable them to form self-assembled structures in aqueous solutions through intermolecular forces, such as micelles, nanosheets, or nanoparticles. As an attractive nano-drug, carrier-free self-assembly has been widely studied because of its high biological safety, high drug loading, simple drug components, and simplified synthesis procedures [4,5,6].

Traditional Chinese medicine (TCM) is one of the treasures of the Chinese nation, which has great advantages in the clinical treatment of many diseases [7,8]. In recent years, carrier-free self-assembly has gradually shifted the focus of research on Chinese medicine, which effectively solves the problems of short half-life, low bioavailability, and poor stability. Self-assembly is an important part of nanotechnology, which widely exists in various fields [9,10]. Self-assembly of phytochemicals originated from TCM is a hot topic in recent years. TCM has complex composition and unique structure, and thus its components are prone to interact to produce aggregation or self-assembly to form aggregates [11,12].

Rhein is a main active ingredient in Rhei Radix et Rhizoma (Da Huang), a traditional Chinese medicine. Rhein is known for its poor solubility in water, which presents challenges for its use in pharmaceutical applications and biological studies. The solubility of a compound is crucial for its absorption, distribution, metabolism, and excretion (ADME) in the body. Poorly soluble compounds tend to have limited bioavailability, which can significantly hinder their therapeutic effectiveness [13,14,15]. The low water solubility of Rhein is attributed to its chemical structure, which is characterized by a rigid aromatic anthraquinone backbone that lacks polar functional groups capable of forming hydrogen bonds with water molecules. This property makes it difficult for Rhein to dissolve in aqueous environments. However, self-assembly of Rhein has made some progress in recent years. For example, Rhein can directly self-assemble into 3D fiber hydrogels [16], and the preparation of nanopreparations is possible by self-assembly of Rhein and doxorubicin molecules as emulsifiers [17]. Thus, previous studies show that self-assembly of Rhein has the potential in the fields of drug delivery and biomaterials. Matrine is a natural compound that has gained significant attention in the fields of medicine and pharmacology. It possesses a wide range of pharmacological activities. One of its notable effects is its anti-inflammatory properties. It can help regulate the body’s inflammatory response, which is beneficial in treating various inflammatory disorders [18]. Studies have indicated its potential in modulating the immune system, enhancing the body’s defense mechanisms. However, like many natural compounds, the application of Matrine requires further research and clinical trials to fully understand its efficacy, safety, and optimal dosage for different conditions. A recent study reported the self-assembly of encapsulated zinc (II) phthalocyanine Matrine-PROTAC self-assembly of phthalocyanine Matrine-PROTAC for cancer therapy [19]. However, further research and development are needed to realize their wide application.

**Figure 1:**
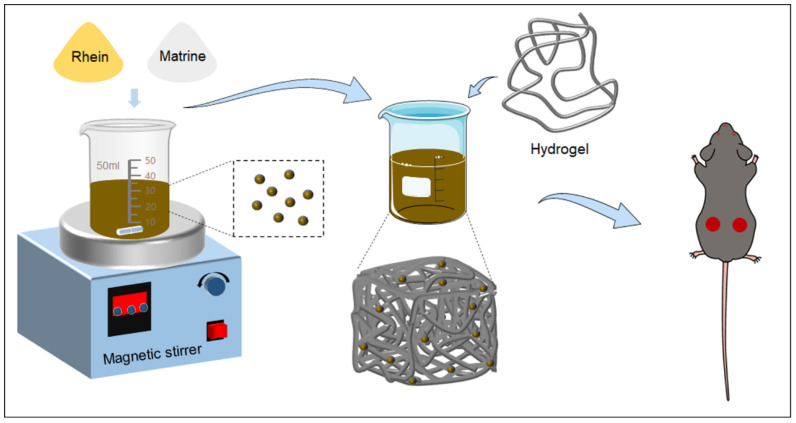
The schematic illustration the self-assembly of Rhein and Matrine into GelMA hydrogel with wound-healing application. Firstly, Rhein and Matrine were mixed in a certain proportion and heated on a magnetic stirrer to synthesize self-assembled nanoparticles. Then, the self-assembled nanoparticles were added into the hydrogel to formulate the hydrogel. Finally, a mouse wound-healing model was established to investigate the therapeutic effects of the drug-containing hydrogel on wound healing in mice.

Wound healing is a complex and dynamic process that has seen significant advancements in recent years. Numerous investigators have focused on the development of innovative approaches for therapeutic angiogenesis to aid in healing of chronic wounds. Currently, many techniques have been applied in improving wound healing, including the delivery of growth factors, gene therapy, stem-cell therapies and mechanical-based stimulation [20,21,22,23]. For instance, nanofiber scaffolds have shown great potential as they offer high porosity and surface area, facilitating cell adhesion and proliferation. Advances in stem-cell research have also had a major impact on wound-healing treatments. Mesenchymal stem cells have been explored for their ability to secrete growth factors and cytokines that promote wound closure and tissue repair. Experiments have demonstrated that transplanting these stem cells into wounds can accelerate the healing process and improve the quality of the healed tissue. Novel biomaterials with integrated pharmacologic and tissue regenerative functions are typically biodegradable and include microporosity to allow for vascularization and cell recruitment [23]. In addition, herbal remedies have been helpful in the treatment of dermatological disorders such as skin lesions, eczema, burns, and hypertrophic scars [22]. However, the poor solubility of asiaticoside in aqueous medium is a major limiting factor in the drug’s therapeutic efficacy [24]. To improve the pharmacological effect of free asiaticoside, carriers have been developed to increase wound-healing potential. Herein, we designed a carrier-free self-assembly delivery system, assembled from Rhein and Matrine through hydrogen bonding and π−π stacking interactions. Furthermore, the molecular mechanisms of self-assembly were explored. In addition, a RM NP hydrogel was developed and applied in wound-healing mice (Figure 1). In brief, the self-assembly technology of small molecules in TCM provides a new strategy for improving the efficacy and safety of TCM, as well as new opportunities for the modernization and internationalization of traditional TCM. However, in-depth studies on the self-assembly process, biodistribution, metabolism, and pharmacodynamics are needed to translate these advantages into practical clinical applications.

## 2. Results

### 2.1. Preparation and Characterization of RM NPs

**Figure 2:**
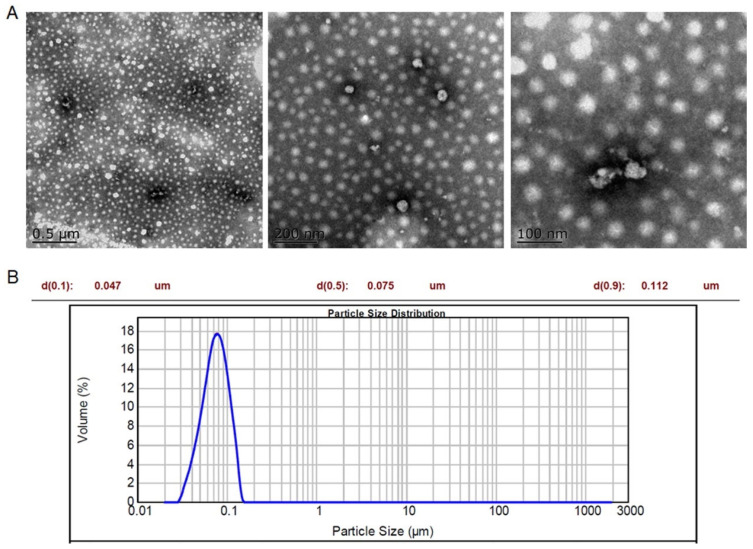
Microscopic morphology of self-assemblies. (**A**) SEM characterization of RM NPs. (**B**) The average diameter of the RM NPs.

Herein, we found Rhein and Matrine could self-assemble into nanoparticles without any involvement of carriers and excipients. Meanwhile, Matrine was also demonstrated as an anti-inflammatory and antibacterial ingredient, which might result in a synergistic effect with Rhein. Thus, a carrier-free and biocompatible self-assembly system was constructed. Rhein and Matrine were self-assembled into nanoparticles with the molar ratio of the amount of substance of 1:1. Firstly, transmission electron microscopy (TEM) was used to characterize the morphology of the RM NPs, and results showed that the RM NPs were close to spherical (Figure 2A). Furthermore, the sizes of the RM NPs were characterized by dynamic light scattering (DLS). The DLS results showed that the average size of nanoparticles was approximately 75 nm (Figure 2B). These show the successful self-assembly of Rhein and Matrine. Self-assembly is the process by which molecules, nanoparticles, or other building units spontaneously form ordered structures through interactions without external intervention. This bottom-up assembly method has a wide range of applications in fields such as materials science, chemistry, and biology.

### 2.2. Formation of RM NPs

**Figure 3:**
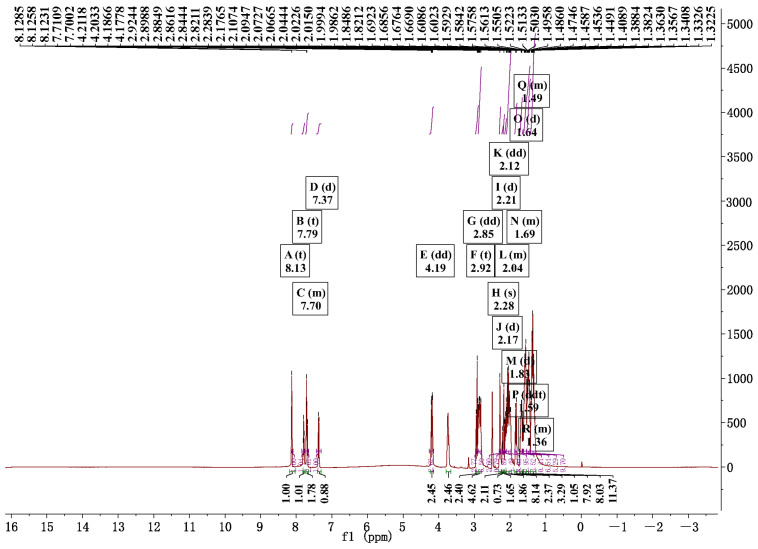
^1^H NMR spectrum of RM NPs. ^1^H NMR (500 MHz, DMSO) δ 8.13 (t, *J* = 1.3 Hz, 1H), 7.79 (t, *J* = 7.9 Hz, 1H), 7.73−7.66 (m, 2H), 7.37 (d, *J* = 8.3 Hz, 1H), 4.19 (dd, *J* = 12.7, 4.3 Hz, 3H), 2.92 (t, *J* = 12.7 Hz, 3H), 2.85 (dd, *J* = 20.3, 11.6 Hz, 6H), 2.28 (s, 3H), 2.21 (d, *J* = 5.1 Hz, 1H), 2.17 (d, *J* = 5.0 Hz, 2H), 2.12 (dd, *J* = 10.6, 5.6 Hz, 1H), 2.10−1.97 (m, 8H), 1.83 (d, *J* = 13.7 Hz, 3H), 1.73−1.66 (m, 4H), 1.64 (d, *J* = 3.9 Hz, 0H), 1.59 (ddt, *J* = 16.9, 12.6, 4.7 Hz, 7H), 1.53−1.43 (m, 5H), 1.43−1.28 (m, 10H).

To further confirm the weak interaction types and positions between Rhein and Matrine, the ^1^H NMR of RM NPs was measured in dimethyl sulfoxide-d6 and then compared with the monomer Rhein and Matrine under the identical conditions. Representative ^1^H NMR spectra showed that the RM NPs had the H signals of Rhein and Matrine, illustrating the successful synthesis of the conjugated RM NPs (Figure 3 and Figure S1). It was obvious that the chemical shifts of the H signals in Rhein moved up-field systematically from 7.39, 7.73, 7.81, and 8.12 ppm to 8.13, 7.79, 7.70, and 7.37 ppm after Rhein and Matrine formed nanoparticles (Figure 3, Figures S1 and S2). After the self-assembly of RM NPs, the chemical shifts of H peaks in Matrine also changed apparently from 4.18, 3.71, 2.90, 2.72, 2.14, 2.00, 1.85, 1.70, 1.55, 1.41, and 1.31 ppm to 4.19, 2.92, 2.85, 2.28, 2.17, 2.12, 2.04, 1.83, 1.69, 1.59, and 1.36 ppm. The H signals on the Matrine ring, which were involved in the conjugation of Rhein, had an obvious shift. The fact was that the anthracene ring in the Rhein and Matrine became a structure of π−π stacking, which enhanced the delocalization of electrons and made the electronic fraction more extensive. To summarize, it proved that Rhein and Matrine could self-assemble in hydrofacies.

**Figure 4:**
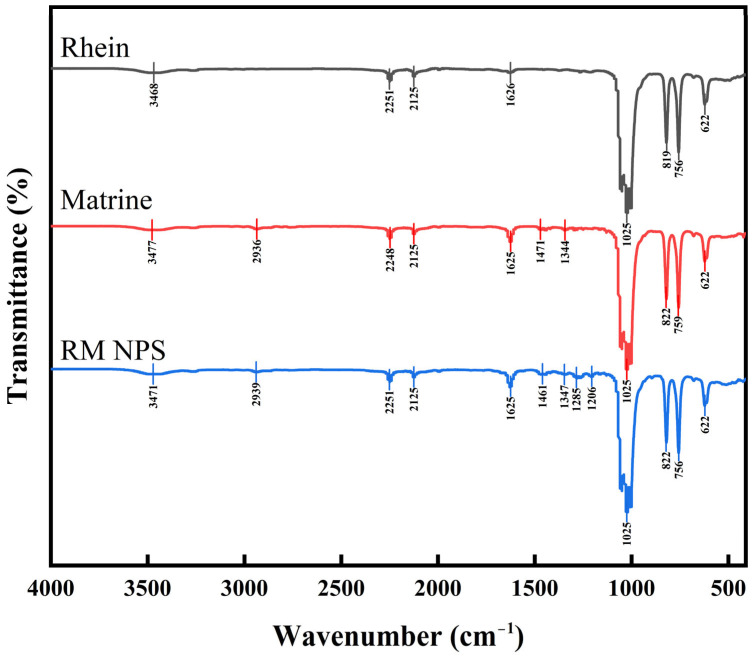
FT−IR spectra of Rhein, Matrine, and RM NPs.

The Fourier transform infrared spectra (FT-IR) of nanoparticles provided more information for the interaction between Rhein and Matrine. After forming RM NPs, it can be seen that the stretching vibration peak at 3471 cm^−1^ corresponding to the hydroxyl group (O-H) in Rhein or the amine group (N-H) in Matrine, which has a broadened peak band, indicates that the O-H and N-H groups are involved in hydrogen bonding during the self-assembly process; 2938 cm^−1^ corresponding to the C-H stretching vibration peaks of the Rhein and Matrine molecules correspond to the C-H stretching vibration peaks of methyl and methylene (CH_3_ and CH_2_) in Rhein and Matrine molecules; the C=O stretching vibration peaks at 1625 cm^−1^ correspond to the amide group (-CONH-) in RM NPs, which originates from the amide group contained in Matrine; the stretching vibration peaks at 1347 cm^−1^ and 1285 cm^−1^ correspond to the C-N bonding in the Matrine molecule and C-O bonding in the Rhein molecule; the stretching vibration peaks at 1206 cm^−1^ and 1025 cm^−1^ correspond to the stretching vibration peaks of C-O and C-N bonds in RM NPs. Rhein contains hydroxyl and ether bonds, and Matrine contains C-N bonds; the stretching vibration peaks at 822 cm^−1^ and 756 cm^−1^ are related to the C-H outward bending vibration of aromatic rings in RM NPs; Rhein is an anthraquinone derivative and contains multiple aromatic rings; 622 cm^−1^ corresponds to the C-H out-of-plane bending vibration peak of the ring structure in the RM NP molecule (Figure 4). These variations suggest that RM NPs are binary aggregates composed of Rhein and Matrine that form stable complexes through hydrogen bonding and other intermolecular interactions.

### 2.3. Molecular Dynamics Simulation Analysis of RM NPs

**Figure 5:**
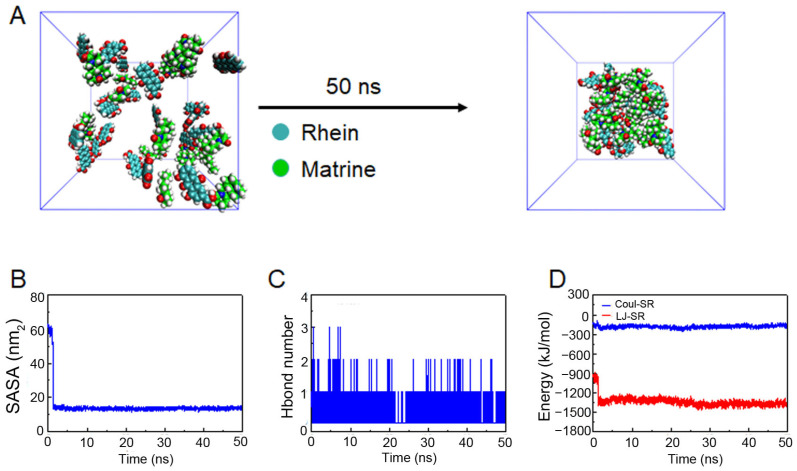
Intermolecular interaction modes between Rhein and Matrine. (**A**) Structural changes in the initial and final states of the Rhein and Matrine system during simulations. Red balls indicate hydroxyl group (−OH). (**B**) Variation of solvent-accessible surface area with simulation time. (**C**) Variation of H-bond number with simulation time. (**D**) Changes in intermolecular binding energy with simulation time.

Through the previous ^1^H NMR analysis, we have demonstrated strong and weak changes in the interaction energies during the self-assembly of different molecular systems. In order to deeply study the differences in self-assembly, the conformations of MD simulation were analyzed. A shown in Figure 5A, after 50 ns of MD simulation, all the small molecules in the Rhein and Matrine system formed a relatively stable, dense sphere-like nanocluster structure. As the nanoparticles proceed in self-assembly, the area exposed to the solvent environment gradually decreases, so the solvent accessible surface area (SASA) can be used to evaluate the degree of compactness of the nanoparticles. The relative changes of the SASA of the system during the simulation process are shown in Figure 5B. In short, the SASA value was decreased to different degrees during the simulation process, indicating that different degrees of aggregation phenomena occurred in the system during the simulation process. Specifically, the SASA values of the system showed an obvious decrease in the initial stage of the simulation, and then the decrease slowed down and stabilized. Since both Rhein and Matrine molecules contain hydrogen bond donors and acceptors, the hydrogen bonding effect may be one of the important factors for the intermolecular interaction and aggregation. In order to further investigate the driving force in the interaction and aggregation process of the system, the total number of hydrogen bonds between Rhein and Matrine were statistically analyzed. As shown in Figure 5C, the variation of the number of hydrogen bonds between Rhein and Matrine during the self-assembly process with simulation time was analyzed. The results show that the number of hydrogen bonds stabilizes immediately, indicating that the two molecules have already aggregated to form a relatively stable nanocluster structure.

In order to further investigate the driving force in the process of self-assembly of the system into nanoparticles, herein, the interaction energies between representative molecules were analyzed. Coul-SR represents electrostatic energy, and the main types of interactions are hydrogen bonding or ionic bonding; LJ-SR represents van der Waals energy, and the main types of interactions are various conjugation interactions. In the RM NP system, the average value of Coul-SR is −183.060 ± 19.717 kJ/mol, and the average value of LJ-SR is −1342.671 ± 69.643 kJ/mol (Figure 5D). Comparison of the binding energies between the self-assembled molecules reveals that the electrostatic and van der Waals energies are comparable, and the stronger interaction energies indicate that the molecules are able to self-assemble to form compact nanoclusters.

### 2.4. Intermolecular Interaction Modes of RM NPs

**Figure 6:**
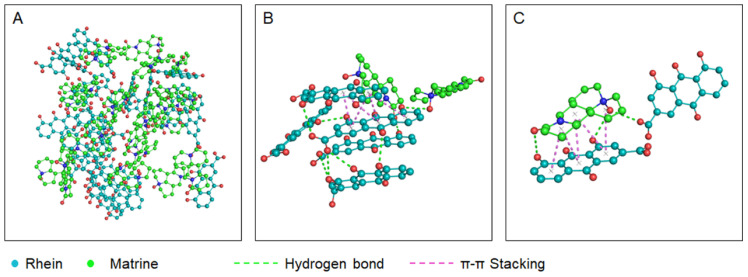
Patterns of interactions between Rhein and Matrine molecules. (**A**) Patterns of interactions between Rhein and Matrine molecules in complex systems. (**B**) Enlarged image: Hydrogen bonding interactions are formed between the hydroxyl group on the ring of the Rhein molecule and the oxygen atom on the ring of the Matrine molecule, and intramolecular hydrogen bonding interactions are formed between the hydroxyl group and the carbonyl oxygen atom inside the Rhein molecule. (**C**) Enlarged image: Rhein molecules also bind to Matrine molecules through π−π stacking. Red balls indicate hydroxyl group (−OH).

In addition, the hydroxyl group on the ring of the Rhein molecule forms hydrogen bonding with the oxygen atom on the ring of the Matrine molecule. Furthermore, the hydroxyl group inside the Rhein molecule forms an intramolecular hydrogen bond with the carbonyl oxygen atom, and the Rhein molecule binds to the Rhein molecule through π−π stacking. With the π−π stacking, intermolecular hydrogen bonding and intramolecular hydrogen bonding interactions are mainly promoted between Rhein molecules and Matrine molecules (Figure 6).

### 2.5. Characterization of GelMA Hydrogel Loaded with RM NPs

**Figure 7:**
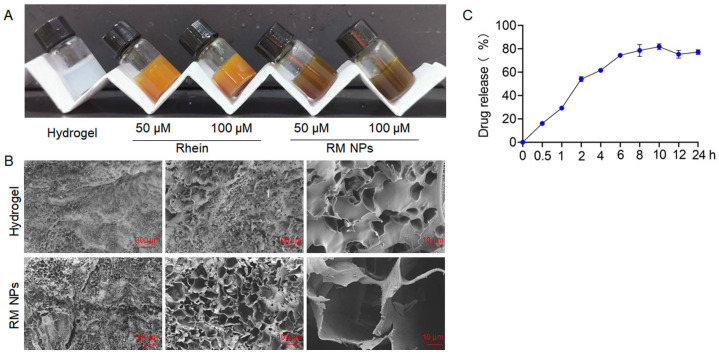
Physicochemical characterization of blank hydrogel, Rhein-loaded hydrogel and RM NPs-loaded hydrogel. (**A**) Photographs of blank hydrogels and hydrogels with different contents of Rhein and RM NPs. (**B**) The scanning electron microscope (SEM) micrographs of blank hydrogel and RM NPs-loaded hydrogel. (**C**) The release of RM NPs in PBS (pH = 7.4).

Gelatine Methacrylate (GelMA) hydrogel is a photo-crosslinked hydrogel synthesized from gelatin and methacrylic anhydride. It has been widely used in the fields of bone-tissue engineering, drug delivery, and three-dimensional cell culture because of its good biocompatibility, adjustability, and injectability [25,26]. Herein, GelMA hydrogel was used as a drug carrier for controlled release and targeted delivery of RM NPs. It can control the release rate and release time of RM NPs in the gel to improve the efficacy of the drug. Rhein and RM NPs were dispersed uniformly in the hydrogels, as shown in Figure 7A; with different primary amounts of Rhein and RM NPs, the color of the hydrogels gradually deepened. Additionally, the GelMA hydrogels loaded with RM NPs can hold their shapes without collapse after freeze-drying (Figure 7B). SEM images showed that the hydrogels possessed a three-dimensional, interconnected, and porous structure after freeze-drying (Figure 7B). After RM NPs were introduced into the hydrogel, the pore size of the hydrogel was increased. This might increase the softness of the hydrogel and increase efficacy. In terms of the release and stability characteristics of RM NPs, after 24 h of dialysis in PBS (37 °C), about 80% of the RM NPs were released, indicating that the stacking structure could be sustainably released in a physiological environment (Figure 7C).

### 2.6. Effect of RM NPs on Wound Healing in Mice

**Figure 8:**
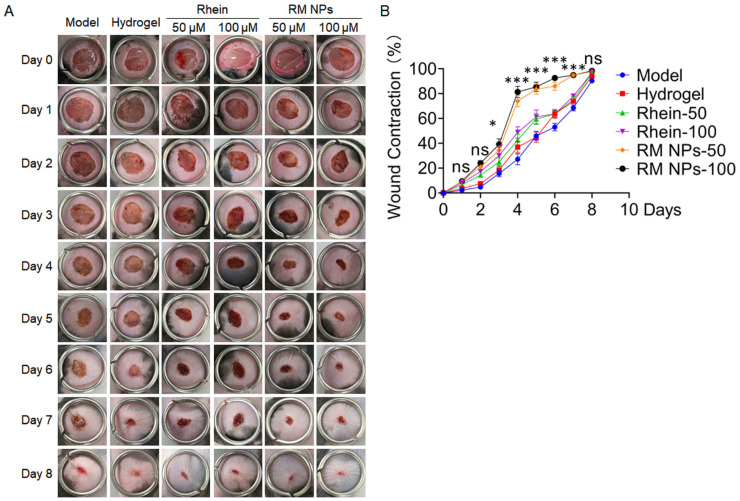
Optical photographs of wound-bed recovery covered by blank hydrogel, hydrogel with Rhein, or RM NPs for 9 days. (**A**) Photographs of wounds treated with different treatments on days 0, 1, 2, 3, 4, 5, 6, 7, 8, and 9; (**B**) Wound-healing rate of wounds in mice (*n* = 6). The values are the mean ± SD. ns represent no significance, * *p* < 0.05, *** *p* < 0.001, RM NPs-treated group compared to the Rhein group.

The potential wound repair properties of Rhein and RM NPs-loaded hydrogels were evaluated on a skin wound-healing model. Wounds (diameter of 1 cm) were created on the backs of mice. Non-treated wounds were set as the model group, and blank hydrogel was set as control group. Rhein dispersion (50 μM and 100 μM) and RM NPs dispersion (50 μM and 100 μM) were set as the treated group. After different treatments, the wounds of mice were photographed every day after surgery. As shown in Figure 8, in contrast with the untreated model group, blank hydrogel, the Rhein-loaded hydrogel and RM NPs-loaded hydrogel could accelerate skin-wound healing obviously. These results indicated that the application of hydrogels significantly accelerated wound healing during the healing process. In particular, contrast with the Rhein-treated group, RM NPs-loaded hydrogels presented better protection effects against skin damages.

## 3. Discussion

Many herbal components are not easily absorbed by human body due to poor water solubility and low biofilm permeability [14]. The formation of nanoparticles by self-assembly can significantly improve the solubility and bioavailability of these herbal ingredients, thus enhancing their therapeutic effects. Self-assembled nanostructures can protect small molecules from being damaged by the external environment, such as degradation by light, heat, or enzymes, thus enhancing their chemical stability and the drug’s effective period [12,13]. Herein, our study presents a novel approach by focusing on the self-assembly of Rhein and Matrine nanoparticles for enhanced wound healing. Unlike previous research that may have investigated these compounds separately or in different formulations, this work demonstrates their unique combination and self-assembly. This self-assembly leads to distinct physicochemical and biological properties that have not been previously explored. Specifically, after self-assembly and formation of nanoparticles, the physicochemical properties of Rhein are changed, and its solubility is significantly increased (Figure S3). Improved solubility allows more Rhein molecules to interact with its target, thus enhancing its effect. In addition, the synergistic effect between the two compounds should not be overlooked. This synergy may be manifested on several levels, such as complementing or enhancing the mechanism of action. In addition, the synergistic effect may also involve a synergistic modulation of intracellular signaling pathways, affecting cellular physiological processes, which in turn may have a more positive impact on the relevant condition. Further in-depth study of the specific mechanisms and aspects of such synergistic effects will help us to better understand and optimize the application of self-assembled nanoparticles of Rhein.

In order to gain a deeper understanding of the effects, different ratios of Rhein and Matrine on self-assembly need further exploration. To further explore the self-assembly mechanism, a variety of research methods can be used, such as experimental studies, computer simulations, and theoretical analyses [14,27]. Experimental studies can directly observe and characterize the self-assembly structure, providing visual evidence [28]. Computer simulations can help us predict the self-assembly behavior at different ratios and reveal the underlying mechanisms. Theoretical analysis, on the other hand, can provide a deeper understanding and explanation of the self-assembly phenomenon [14,27]. In this study, the 1:1 ratio (molar ratio) was explored. However, different ratios may affect the patterns and morphology of self-assembly. For example, in polymer self-assembly, changing the molecular weight distribution, the ratio of blocks, or the ratio of functional groups of a polymer can lead to different self-assembled structures, such as lamellar, columnar, and spherical. By changing the ratios of different components, the physical and chemical properties of the self-assembled system may be modulated, thus realizing precise control of the self-assembly mode and morphology [18,27]. Thus, the effects of different ratios on self-assembly patterns and morphology will be further explored. In conclusion, exploring the effects of different ratios on self-assembly patterns and morphology is a research direction of great significance. By studying this issue in depth, we can better control and design self-assembled materials and provide more possibilities for their applications in various fields.

The primary focus of our study was on the wound-healing properties of the composite. Thus, we just assessed the efficacy of the RM NPs in promoting tissue regeneration and repair within the wound environment. However, Matrine and Rhein are compounds that have shown potential antibacterial effects against both gram-positive and gram-negative bacteria, although their mechanisms and efficacies may vary. However, the effects of RM NPs as antibacterials are still unknown [29,30]. Moreover, while they show potential, their use as standalone antibacterial agents may have limitations, and further research is needed to optimize their efficacy and explore combination therapies. Additionally, combination with other antibiotics or compounds may enhance their antibacterial effects and overcome potential resistance mechanisms. In conclusion, although Matrine and Rhein hold promise in the fight against both gram-positive and gram-negative bacteria, continued in-depth research is necessary to fully understand their mechanisms of action and to develop effective therapeutic strategies based on their antibacterial properties. We recognize the potential significance of investigating the antibacterial activity of the composite in future studies. Such an analysis could provide additional valuable insights into the multifunctional capabilities of the material and its overall potential for wound management. We will consider including these antibacterial activity tests in our future research to further expand and complete the understanding of the composite’s performance and applications. In addition, to better understand the effects of nanoparticles on wound healing, the downstream molecular mechanisms need further exploration. This may include studying aspects of how nanoparticles affect cell signaling pathways, gene expression, and protein synthesis. For example, we can analyze the changes in genes and proteins in cells after nanoparticle treatment by using gene microarrays or proteomics techniques to determine which molecular pathways are activated or inhibited.

The skin, as the largest organ in the body, is often damaged by disease, burns, accidental trauma, and surgery. Delayed healing of skin wounds not only affects the integrity of the skin, but it may also lead to infection and scar formation [31,32]. Therefore, shortening the healing time of skin wounds after injury and restoring the skin’s structural and functional integrity are urgent clinical problems. Wound healing is a complex biological process involving multiple stages of hemostasis, inflammation, proliferation, and remodeling. Among these, neovascularization, the formation of new blood vessels, is critical for delivering oxygen and nutrients to the wound site, thereby promoting tissue repair and regeneration. The efficiency of neovascularization significantly affects the duration and outcome of wound healing, and therefore the development of therapeutic strategies to promote neovascularization is not only expected to prevent scar formation, but also to facilitate the skin’s repair process. However, some shortcomings still exist in this study. Herein, the pharmacological effects of nanoparticles were studied. The exploration of a molecular mechanism is lacking, which is a future research direction [31]. Another important aspect is to study the distribution and metabolism of nanoparticles in vivo. In addition, to assess their long-term safety and efficacy, the metabolic pathways and clearance mechanisms of nanoparticles also need to be studied [32].

## 4. Materials and Methods

### 4.1. Reagents

Rhein and Matrine (purity ≥ 98%) were obtained from Sichuan Cuiyirun Co., Ltd (Chengdu, China). Isoflurane (#I5627) was purchased from Sigma-Aldrich (St. Louis, MO, USA). Dimethyl sulfoxide-*d_6_* (D, 99.8%) TMS (0.03%) was purchased from Energy Chemical Co., Ltd. (Shanghai, China). GelMA hydrogel and 0.25% lithium phenyl-2,4,6-trimethylbenzoylphosphinate (LAP) solution were obtained from Engineering For Life (Zhejiang, China).

### 4.2. Preparation of RM NPs

Rhein and Matrine NPs were prepared by a one-step self-assembly process according to the references with slight modification [9]. Briefly, Rhein and Matrine were mixed in a beaker (molar ratio, 1:1) in a dimethyl sulfoxide (DMSO) solution under vigorous stirring and heating at 60 °C for 6 h. Then, the solution in a dialysis bag (MWCO = 2.5 kDa) was dialyzed against ultrapure water for 12 h. Finally, the self-assembled RM NP suspension was obtained.

### 4.3. Characterization of the RM NPs

The morphology of RM NPs was imaged on a TEM microscope (Hitachi HT7700 TEM, HITACHI, Tokyo, Japan). The morphology of the hydrogel was imaged using scanning electron microscopy (SEM, ZEISS-sigma 300, Zeiss, Jena, Germany). The hydrodynamic diameters of RM NPs were measured using dynamic light scattering (Mastersizer 2000, Malvern, UK).

### 4.4. Molecular Dynamics Simulation Analysis of RM NPs

A 6 nm × 6 nm × 6 nm simulation box was first constructed and randomly filled with 20 Rhein and 20 Matrine molecules. Molecular dynamics (MDs) simulation was performed using the Gromacs 2018.4 program at constant temperature and pressure [33]. The GAFF all-atom force field, TIP3P water model, was applied [34]. During MD simulations, all involved hydrogen bonds were constrained using the LINCS algorithm with an integration step of 2 fs [35]. Electrostatic interactions were calculated using the (Particle-Mesh Ewald) PME method [36]. The non-bonded interaction cutoff was set to 10 Å and updated every 10 steps. The V-rescale temperature coupling method was used to control the simulation temperature to 298.15 K [37]. The Parrinello–Rahman method was used to control the pressure to 1 bar [38]. The steepest descent method was used to minimize the energy of the four systems to eliminate the too-close contact between the atoms; then, NVT equilibrium simulations were performed for 100 ps at 298.15 K. Finally, MD simulations were performed for 50 ns for each of the 2 different systems. The conformations were saved every 10 ps, and the visualization of the simulation results was performed using the Gromacs embedded program and VMD.

### 4.5. Preparation and Characterization of GelMA Hydrogels Loaded with RM NPs

GelMA hydrogel was dissolved in 0.25% lithium phenyl-2,4,6-trimethylbenzoylphosphinate (LAP) solution (Engineering For Life, Zhejiang, China). Then, different concentrations of Rhein and RM NPs (final concentration at 50 and 100 μM) were added to the hydrogel. After that, hydrogel constructs were photo-crosslinked by exposure to 405 nm UV light for 25–30 s.

### 4.6. Drug-Release Test

For studying the release behavior of RM NPs loaded in hydrogel, PBS solution of RM NPs loaded in hydrogel (100 µmol/L, 1 mL) in a dialysis bag (MW = 3.5 kDa) was immersed into PBS (50 mL) at 37 °C. At desired time points (0, 0.5, 1, 2, 4, 6, 8, 10, 12, and 24 h), the dialysis solution (0.5 mL) was collected and analyzed with high-performance liquid chromatography (HPLC). The chromatographic parameters were modified as follows: Acetonitrile-0.1% phosphoric acid solution (85:15) was used as the mobile phase with a detection wavelength of 254 nm, a column temperature of 25 °C, and a flow rate of 1.0 mL/min; each experiment was repeated three times.

### 4.7. Animal

C57BL-6J mice (male, 8–9 weeks) were purchased from Wushi Animal Center (Fuzhou, China). All animal experiments were carried out in accordance with the National Institutes of Health Guide for the Care and Use of Laboratory Animals, following protocols approved by the Pharmaceutical Animal Experimental of Huaqiao University (No. A2021044).

### 4.8. Effect of RM NP Wound Healing in Mice

After anesthetizing the mice with isoflurane, the backs of the mice were depilated. Then a skin-wound model with a diameter of 1 cm was made on the backs of mice. After that, the mice were divided into six groups: model group, hydrogel group, Rhein group (50 μM), Rhein group (100 μM), RM NP group (50 μM), and RM NP group (100 μM). The wounds of mice were photographed every day after surgery, and the wound changes were recorded. The wound areas were measured with ImageJ software (V 1.8.0) to calculate the wound-closure rate of mice in each group.

$$Wound\;close\;rate\left(\%\right)=\frac{\left(A_{0}-A_{n}\right)}{A_{0}}\times 100\%$$

where A_0_, A_n_ represents the wound area of the original and on day n after wounding.

### 4.9. Statistical Analysis

All data were presented as mean ± standard deviation (SD). Statistical significance was assessed using Student’s *t*-test for paired samples and one way ANOVA for multiple group comparisons via GraphPad Prism 8.0 software. A *p*-value of less than 0.05 was considered statistically significant.

## 5. Conclusions

Carrier-free self-assembly is gradually shifting towards natural products, as they significantly enhance bioavailability and drug-loading capacity. Herein, a self-assembled nanostructure of RM NPs was discovered. In addition, the nanoparticles were characterized, and their self-assembly mechanism was further explored. The Rhein and Matrine self-assembly system presents a novel assembly strategy that may offer new directions for the application of natural products.

## Data Availability

The data presented in this study are openly available in article.

## Supplementary Materials

The following supporting information can be downloaded at: https://www.mdpi.com/article/10.3390/molecules29143326/s1, Figure S1: Structural characterization of rhein; Figure S2: Structural characterization of matrine; Figure S3: The Solubility properties of Rhein (left) and RM NPs (right).
