# Supplementary material for: Self-Assembly of Rhein and Matrine Nanoparticles for Enhanced Wound Healing
Source: Molecules. 2024 Jul 15;29(14):3326. doi: 10.3390/molecules29143326 (PMC11279319; doi:10.3390/molecules29143326)
Supplement: Supplementary file 1 [file molecules-29-03326-s001.zip › molecules-3078739-supplementary.pdf]

Supplementary materials

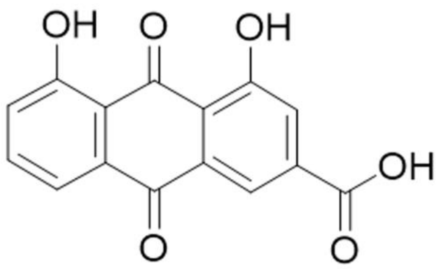

A

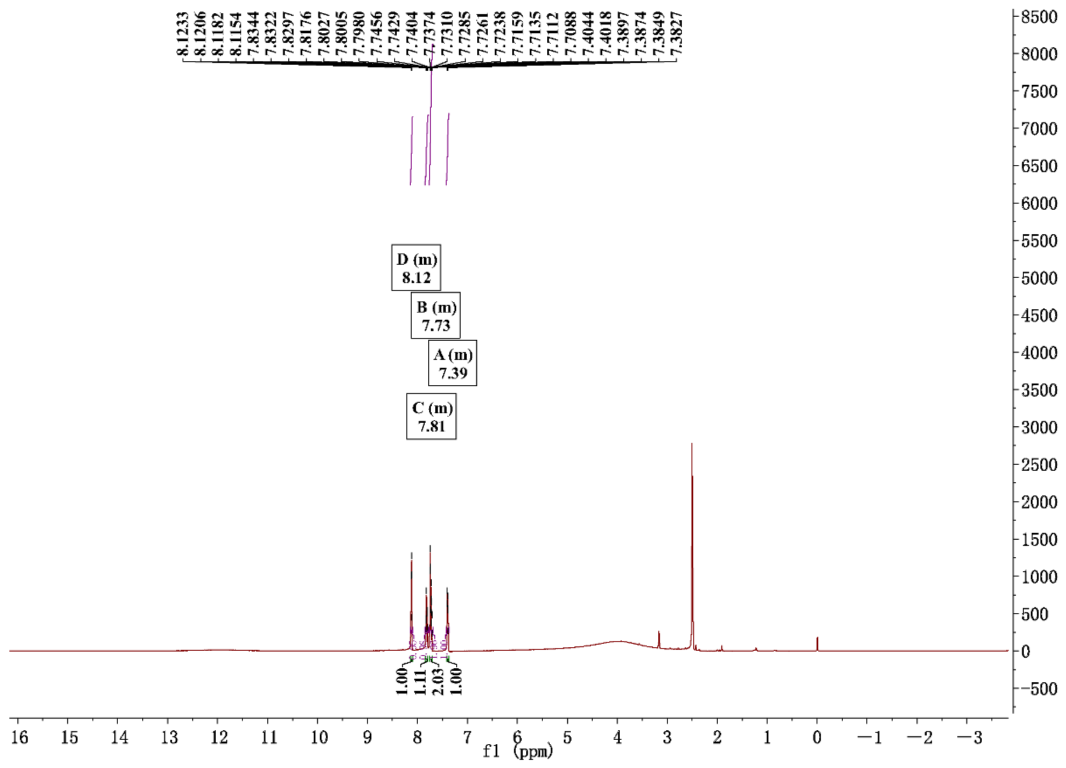

B

Figure S1 Structural characterization of rhein. (A) Structural formula for rhein. (B) 1H NMR spectrum of rhein.

^{1}H NMR (500 MHz, DMSO) δ 8.14 – 8.10 (m, 1H), 7.85 – 7.78 (m, 1H), 7.76 – 7.69 (m, 2H), 7.42 – 7.37 (m, 1H).

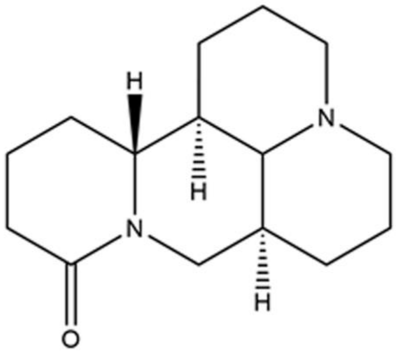

A

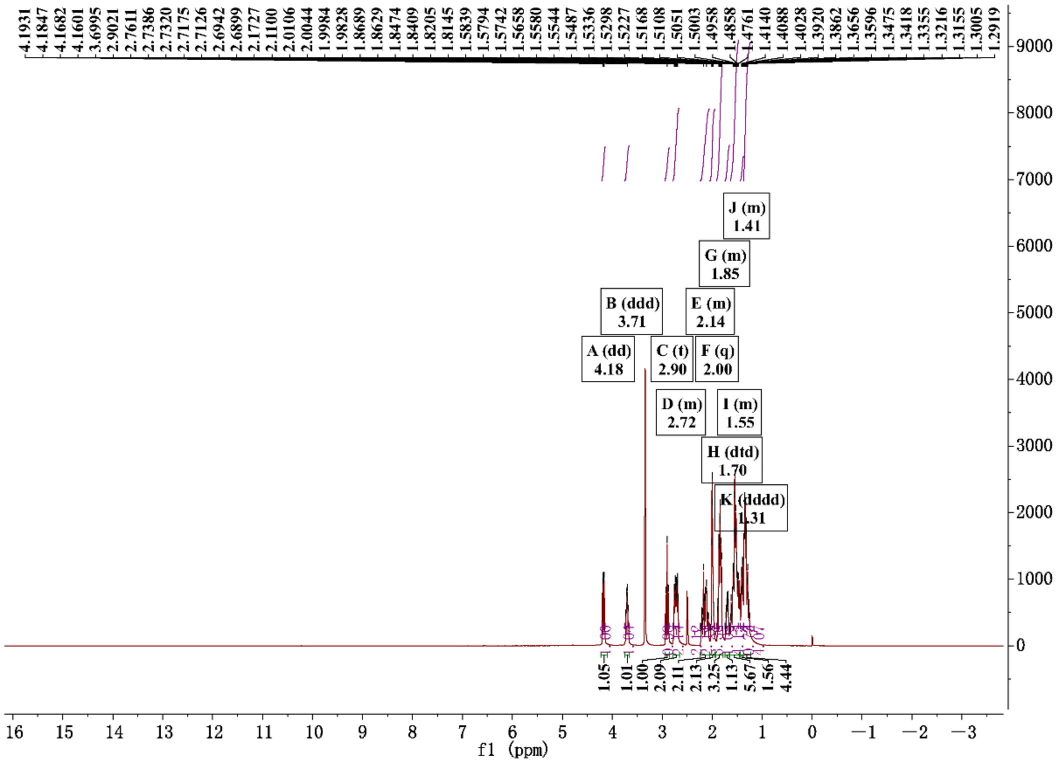

B

Figure S2 Structural characterization of matrine. (A) Structural formula for matrine. (B) 1H NMR spectrum of matrine.

^{1}H NMR (500 MHz, DMSO) δ 4.18 (dd, *J* = 12.3, 4.1 Hz, 1H), 3.71 (ddd, *J* = 10.9, 8.8, 5.7 Hz, 1H), 2.90 (t, *J* = 12.4 Hz, 1H), 2.79 – 2.67 (m, 2H), 2.23 – 2.06 (m, 2H), 2.00 (q, *J* = 5.4 Hz, 2H), 1.91 – 1.80 (m, 3H), 1.70 (dtd, *J* = 10.7, 5.3, 2.7 Hz, 1H), 1.63 – 1.47 (m, 4H), 1.44 – 1.38 (m, 1H), 1.31 (dddd, *J* = 22.4, 18.1, 8.8, 3.6 Hz, 4H).

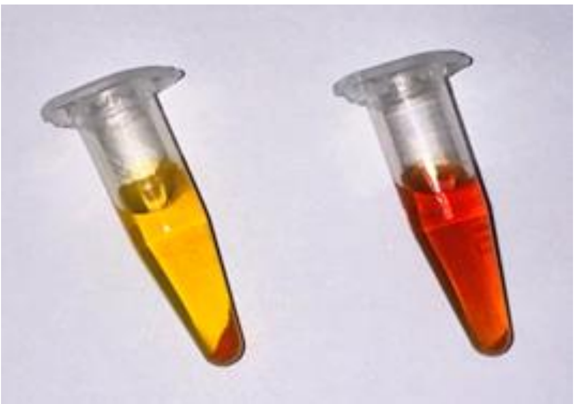

Figure S3 The Solubility properties of Rhein (left) and RM NPs (right).
